# A Functional Genomic Screen for Evolutionarily Conserved Genes Required for Lifespan and Immunity in Germline-Deficient *C. elegans*

**DOI:** 10.1371/journal.pone.0101970

**Published:** 2014-08-05

**Authors:** Amit Sinha, Robbie Rae

**Affiliations:** Department of Evolutionary Biology, Max Planck Institute for Developmental Biology, Tübingen, Germany; Université de Genève, Switzerland

## Abstract

The reproductive system regulates lifespan in insects, nematodes and vertebrates. In *Caenorhabditis elegans* removal of germline increases lifespan by 60% which is dependent upon insulin signaling, nuclear hormone signaling, autophagy and fat metabolism and their microRNA-regulators. Germline-deficient *C. elegans* are also more resistant to various bacterial pathogens but the underlying molecular mechanisms are largely unknown. Firstly, we demonstrate that previously identified genes that regulate the extended lifespan of germline-deficient *C. elegans* (*daf-2*, *daf-16*, *daf-12, tcer-1*, *mir-7.1* and *nhr-80*) are also essential for resistance to the pathogenic bacterium *Xenorhabdus nematophila*. We then use a novel unbiased approach combining laser cell ablation, whole genome microarrays, RNAi screening and exposure to *X. nematophila* to generate a comprehensive genome-wide catalog of genes potentially required for increased lifespan and innate immunity in germline-deficient *C. elegans*. We find 3,440 genes to be upregulated in *C. elegans* germline-deficient animals in a gonad dependent manner, which are significantly enriched for genes involved in insulin signaling, fatty acid desaturation, translation elongation and proteasome complex function. Using RNAi against a subset of 150 candidate genes selected from the microarray results, we show that the upregulated genes such as transcription factor DAF-16/FOXO, the PTEN homolog lipid phosphatase DAF-18 and several components of the proteasome complex (*rpn-6.1*, *rpn-7*, *rpn-9*, *rpn-10*, *rpt-6*, *pbs-3* and *pbs-6*) are essential for both lifespan and immunity of germline deficient animals. We also identify a novel role for genes including *par-5* and T12G3.6 in both lifespan-extension and increased survival on *X. nematophila*. From an evolutionary perspective, most of the genes differentially expressed in germline deficient *C. elegans* also show a conserved expression pattern in germline deficient *Pristionchus pacificus*, a nematode species that diverged from *C. elegans* 250-400 MYA.

## Introduction

The reproductive system in many animals including vertebrates, insects and nematodes regulates lifespan [1]–[13] and the interactions between the reproductive system and lifespan have been extensively studied in *Caenorhabditis elegans*. When the *C. elegans* germline is removed by either genetic means or cell ablation, its lifespan can be extended by 60% [1]. The enhanced lifespan is not a simple life-history trade-off between reproduction and somatic tissue maintenance because *C. elegans* rendered sterile by complete gonad ablation or other manipulations live only as long as *C. elegans* with intact germline [1].

This germline deficient lifespan increase is dependent on an array of genes including the forkhead transcription factor DAF-16/FOXO, nuclear hormone receptors DAF-12 and NHR-80, the cytochrome P450 DAF-9, intestinal FERM domain protein KRI-1, proteasome component RPN-6.1, the microRNAs *mir-71*, *mir-84 and mir-241*, and processes such as insulin signaling, steroid hormone synthesis, increased proteasome activity, autophagy and regulation of fat metabolism [1], [14]–[24]. In addition to increased lifespan, removal of the germline in *C. elegans* and another nematode model organism, *Pristionchus pacificus*, can enhance resistance to bacterial pathogens such as *Xenorhabdus nematophila*, *Serratia marcescens* and *Pseudomonas aeruginosa*, as well as fungal pathogens such as *Cryptococcus neoformans* [2], [25]–[28]. This increase in resistance towards pathogens is also dependent on the FOXO-like transcription factor DAF-16 in both nematodes [2], [26] but varies with pathogen growth conditions [25]. In order to combat bacterial and fungal pathogens *C. elegans* uses various signaling pathways including ERK MAP kinase, p38 MAP kinase, TGF β, JNK-like MAP kinase, the G-protein coupled receptor FSHR-1, bZIP transcription factor *zip-2* and the beta-Catenin/*bar-1* [29]–[36]. In some cases genes involved with lifespan extension also increase pathogen resistance. For example, *C. elegans daf-2* and *age-1* long-lived mutants are also highly resistant to bacteria pathogens [32].

The role of gonadal tissue in regulating pro-longevity or pathogen resistance genes has not been studied at a whole-genome level. Therefore, to understand the contribution of gonadal tissue to lifespan and immunity regulating genes, and the role of the innate immune system towards lifespan extension in germline-deficient *C. elegans* at a genome-wide scale, we combined laser cell ablation to kill the germline and gonad precursor cells, with whole genome microarrays, followed by an RNAi knockdown screen to identify mechanistic processes involved in both longevity and immunity. We also analyzed the evolutionary conservation of germline-regulated genes required for enhanced lifespan and immunity between *C. elegans* and the related model nematode *P. pacificus* [37].

## Results

### Effect of germline and gonad ablation on survival of *C. elegans* exposed to *X. nematophila*

**Figure 1:**
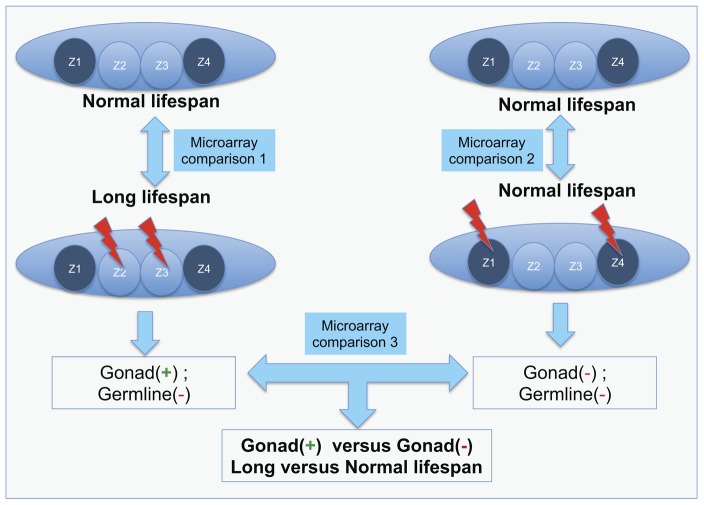
Schematic of germline precursor cells, and cell ablation and microarray experiments. In the *C. elegans* L1 stage larvae, the gonad and germline consist of four cells, namely Z1, Z2, Z3 and Z4 with Z(1,4) producing the gonad and Z(2,3) the germline. If Z(2,3) are killed with a laser beam then only somatic gonad tissue is left, and the animals are long-lived. However if Z(1,4) are ablated then the gonad and the germline are both killed as the germline cannot grow without the influence of the gonad [1] and the lifespan of these animals is comparable to un-ablated controls. The different sets of microarray comparisons as described in the text are also indicated.

**Figure 2:**
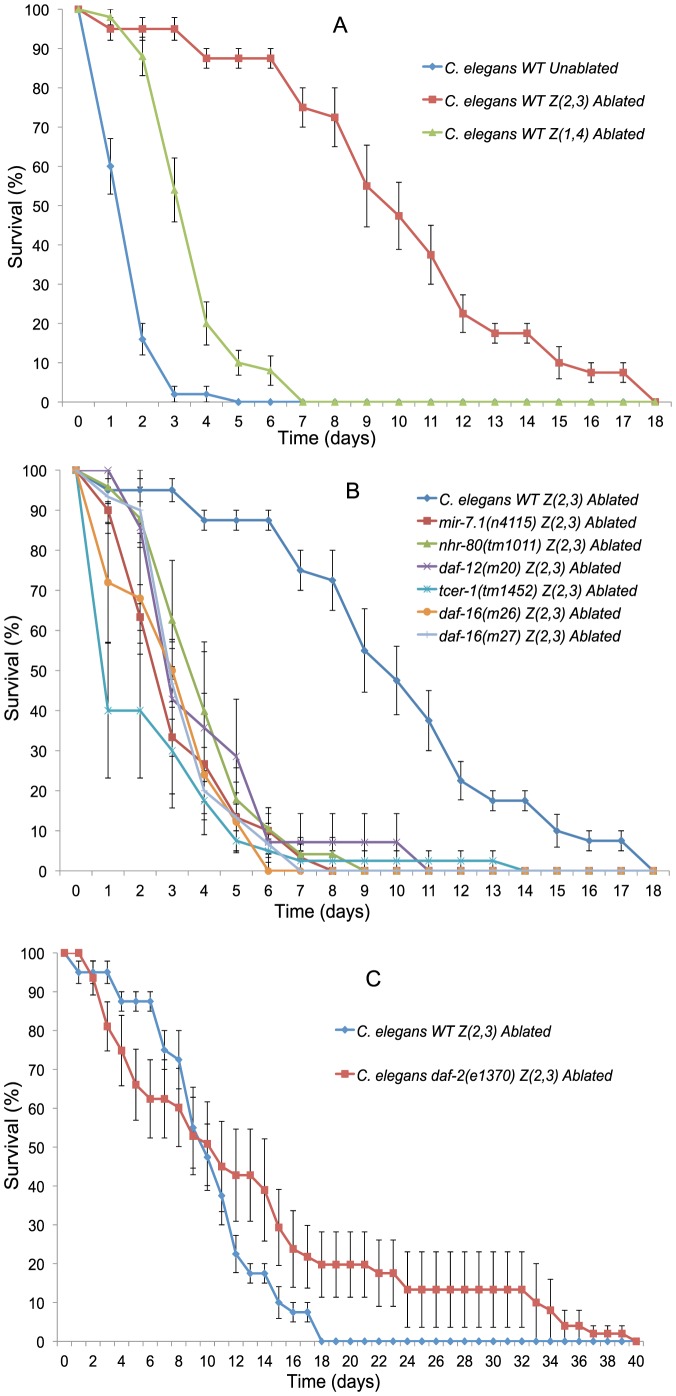
Effect of germline and gonad ablation on the survival of *C. elegans* exposed to pathogenic *X. nematophila*. (A) Survival of *C. elegans* wild-type un-ablated (red) n = 50 (5 independent replicates), Z(2,3) germline-ablated (blue) n = 78 (8) and somatic gonad and germline ablation Z(1,4) (green) n = 48 (5) when exposed to *X. nematophila*. (B) Survival of germline-ablated Z(2,3) *C. elegans* wild-type (dark blue) n = 78 (8), Z(2,3)-ablated *daf-16* (*mu26*) (turquoise) n = 35 (4), Z(2,3)-ablated *daf-16* (*mu27*) (orange) n = 29 (3), Z(2,3)-ablated *daf-16* (*mu86*) (light blue) n = 10 (1), Z(2,3)-ablated *daf-12(m20)* (pink) n = 14 (2), Z(2,3)-ablated *tcer-1(tm1452)* (purple) n = 15 (2), Z(2,3)-ablated *mir-7.1(n4115)* (green) n = 25 (3) and Z(2,3)-ablated *nhr-80(tm1011)* (red) n = 30 (3) when exposed to *X. nematophila*. (C) Survival of Z(2,3)-ablated *C. elegans* wild-type (blue) n = 78 (8) and Z(2,3)-ablated *daf-2(e1370)* mutants (red) n = 47 (5) when exposed to *X. nematophila*.

The formation of the *C. elegans* gonad begins with four precursor cells (Z1,Z2,Z3 and Z4), with Z2 and Z3 giving rise to the germline and Z1 and Z4 making the somatic gonad [38], all of which can be removed using laser microsurgery at the L1 stage (Figure 1). When germline cells Z(2,3) are ablated in *C. elegans* and *P. pacificus* lifespan can be extended by 60% [1], [2]. We wanted to test if the pathogen resistance of *C. elegans* would also be enhanced, as reported in similar studies [2]. We used the bacterium *Xenorhabdus nematophila* as a pathogen, as it is known to be lethal to insects [39] and is also pathogenic to the nematodes *C. elegans* and *P. pacificus* [2], [40]–[42]. Further, transcriptomic profiles of nematodes infected with *X. nematophila* were shown to be highly similar to gene expression profiles induced in *C. elegans* in response to other bacterial pathogens such as *S. marcescens*, *P. aeruginosa* and *Staphylococcus aureus* [42]–[44]. When we fed *C. elegans* with *X. nematophila*, we observed rapid lethality, with all animals dying in one to two days (Figure 2A, see Table S1 for summary statistics), hence we consider *X. nematophila* to be a bona fide nematode pathogen.

When we ablated the germline cells Z(2,3) and fed young-adult *C. elegans* animals *X. nematophila*, we found a significant increase in survival from a mean survival time of 1.8 days in control, un-ablated animals, to 8.5 days in Z(2,3)-ablated *C. elegans* (Figure 2A). However, the ablation of Z(1,4) cells, which also leads to absence of Z(2,3) cells, reduced the mean survival time on *X. nematophila* to 3.7 days (Figure 2A). These results indicate that similar to its effects on longevity [1], the removal of germline contributes to resistance towards bacterial pathogens, potentially via a signal that requires the presence of a functional gonad, a finding that is in accordance with previous observations [25]–[28].

Some of the key genes required for the increase in *C. elegans* lifespan upon the removal of germline precursor cells include *daf-16*, *daf-12*, *tcer-1*, *mir-71* and *nhr-80* [1], [17], [19], [20]. We tested whether these genes would also be required for the increase in the survival of *C. elegans* germline deficient animals on pathogenic *X. nematophilum*, and could show that germline ablations in animals carrying mutations in *daf-16* (*mu26*, *mu27* and *mu86*), *daf-12(m20)*, *tcer-1(tm1452)*, *mir-71(n4115)* and *nhr-80(tm1011)* resulted in a significant decrease in survival when fed *X. nematophila* as compared to germline-ablated wild-type *C. elegans* (Figure 2B). It was previously reported that mutations in insulin-like receptor *daf-2(e1370*) can further increase the lifespan and stress tolerance of germline-ablated animals in *daf-16* dependent manner [1], [45]. We observed a similar synergistic effect of *daf-2(e1370)* mutation and germline ablation on increased pathogen resistance, resulting in further increase in their survival upon *X. nematophila* exposure relative to germline-ablated wild-type *C. elegans* exposed to *X. nematophila* (Figure 2C). These results indicate that genes initially identified as being essential in regulating germline-deficient *C. elegans* lifespan can be essential for combating bacterial pathogens. Hence we can use survival towards pathogens as a rapid tool to identify new genes potentially involved in lifespan regulation.

### Identifying gonad regulated genes in long-lived germline-ablated animals

In order to carry out an unbiased investigation of genes involved in the regulation of lifespan and pathogen resistance, we performed a genome-wide expression analysis of germline-ablated, gonad-ablated and intact *C. elegans* (Figure 1). Since the presence of somatic gonad is essential for the observed increase in lifespan and survival on pathogens (Figures 2A,B,C) [1], [2], [25], [26], we aimed to compare long-lived germline-deficient animals (Z(2,3)-ablated) against the normal-lived germline and gonad-deficient animals (Z(1,4)-ablated), which differ only in the presence of gonadal tissue. We expect this comparison to be more informative and specific for lifespan genes than the comparison between germline-ablated and un-ablated animals because the latter expression profile would be comprised of genes involved not only in lifespan regulation but also include those involved in germline development and maintenance [2], [24], [46]. We employed a common reference experimental design for microarray comparisons (Figure 1) [2], [42], [47]. In the first set of experiments we compared the Z(2,3)-ablated animals against age-matched intact animals as reference, while the second set of experiments compared the Z(1,4)-ablated animals also against intact animals. Since the intact animals used as the reference were common to both experiments, we could compute a contrast between these two experiments to get a list of gonad-regulated genes in long-lived germline-deficient animals (Figure 1).

The first comparison of Z(2,3)-ablated versus intact animals indicated differential expression of 2,794 genes of which 1,587 i.e. about 60% genes were down-regulated (Table S2). This finding is consistent with high transcriptional activity in the germline of intact animals. Further, this set of genes was highly enriched for genes implicated in germline development and function [46], [48] (Table S3). The comparison of Z(1,4)-ablated versus intact animals also showed a trend of predominantly down-regulated genes (more than 95% are down-regulated, Table S4), because gonad ablation takes away the genes expressed not only in the germline but in the gonad as well.

**Figure 3:**
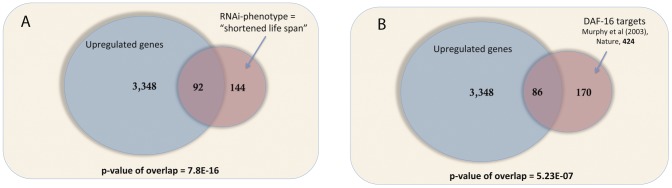
Summary of microarray experiments. (A) Genes up-regulated in the Z(2,3) ablated vs. Z(1,4) ablated comparisons are significantly enriched for genes that are known to shorten lifespan upon knock-down by RNAi. (B) Significant enrichment of DAF-16 targets [51] is observed in the genes up-regulated in Z(2,3)-ablated *vs*. Z(1,4)-ablated comparison.

The comparison of Z(2,3)-ablated *vs*. Z(1,4)-ablated animals is most pertinent for identification of longevity regulating genes. Here we found about 3,440 genes up-regulated while only 150 genes were down-regulated (Table S5). Consistent with the potential role of these genes in lifespan extension, we observed the set of up-regulated genes to be highly enriched for genes with the known RNAi phenotype “shortened lifespan” (p-Value of enrichment  = 7.8E-16, Figure 3A, see Methods). Gene ontology enrichment analysis (Table S6) further identified broad biological processes regulating “determination of adult lifespan”, and “germ cell development”, as well as specific functions such as “translation initiation factor activity”. We also observe enrichment for molecular processes and cellular components involved in “cytochrome-c oxidase activity”, at the “mitochondrial inner membrane” and “mitochondrial envelope”, as well as components of “proteasomal core complex” and “structural constituent of ribosomes”. Searches for significantly enriched gene families revealed various components of proteasomal complex (e.g. *pbs*-, *rpt*-, *rpn*- gene families, Table S7), chaperon activity (*cct*-gene family, Table S7), fatty-acid desaturases (*fat*-gene family, Table S7) and eukaryotic translation elongation factors (*eef*-gene family, Table S7). Together these observations suggest potential roles for proteasome function, translation initiation and elongation factors and lipid metabolism in longevity assurance, consistent with previous reports [17], [20], [22], [49].

### RNAi based screen of candidate genes identifies additional insulin pathway components regulating lifespan and innate immunity

Since many of the genes identified in our microarray experiments could be involved simply in germline and gonadal tissue maintenance rather than in increased longevity and pathogen resistance, we further tested the specificity of these genes for their role in innate immune response and longevity via RNAi. We selected a set of top 100 most upregulated genes plus a random set of 50 genes upregulated at smaller fold-change values, for RNAi based assays (schematic of experiment in Figure S1, genes tested and the corresponding phenotypes observed are annotated in Tables S2-S7). In order to avoid the need for laborious cell ablation of the germline in these RNAi screens, we used the *glp-1*(*e2141*) temperature-sensitive mutant, which does not produce a germline when grown at the restrictive temperature of 25°C [50], and is reported to be long lived and resistant to bacterial pathogens [25]–[28]. We assume here that there are only subtle differences between rendering *C. elegans* germline-deficient via either cell ablation or *glp-1* mutation. Any RNAi candidates that reduced survival of the *glp-1(e1241)* when fed *X. nematophila* were also tested for their effects on normal lifespan of *C. elegans glp-1(e1241)* mutant, and wild type *C. elegans* (see Fig S2, S3 Table S8-S10).

**Figure 4:**
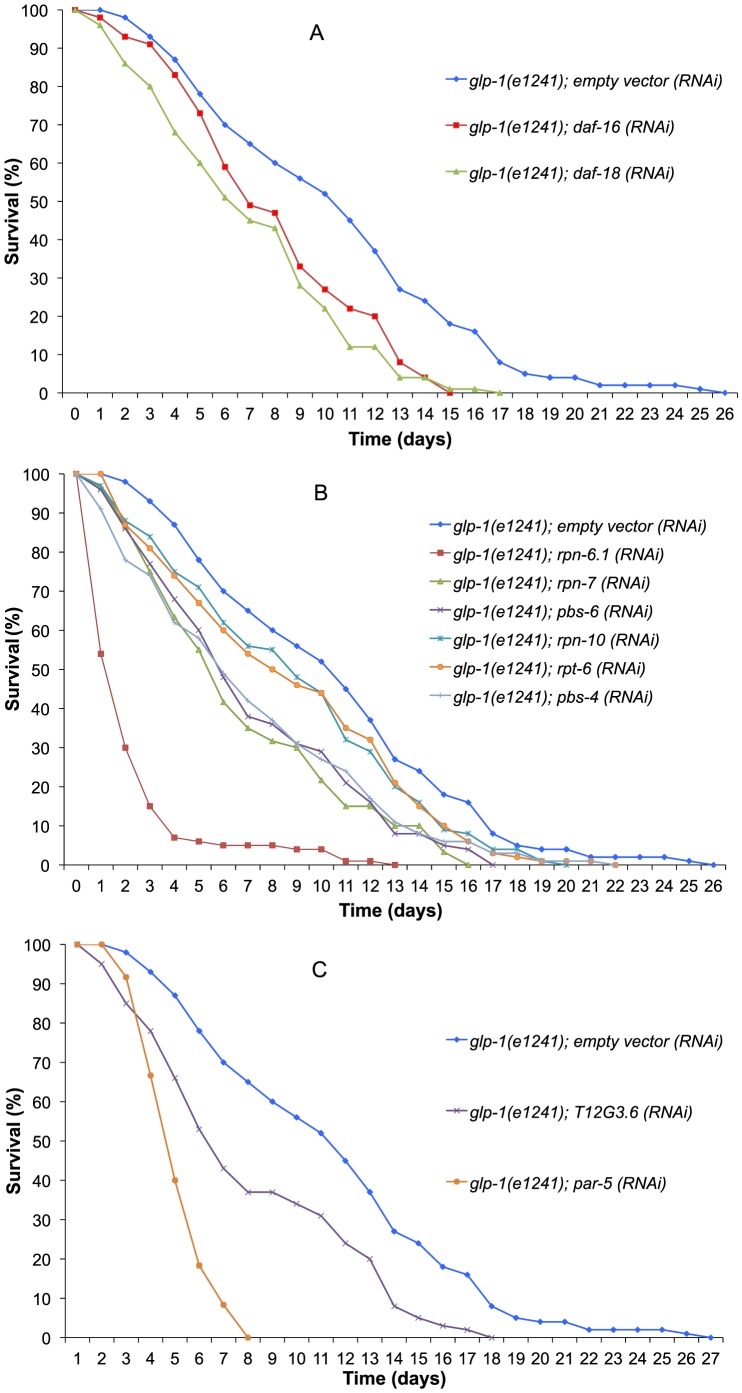
RNAi screening for genes integral for survival towards *X. nematophila* from genome wide analysis. (A) The effect of insulin signaling genes identified as being integral for survival towards *X. nematophila* from genome wide analysis. Survival of *C. elegans glp-1(e2141)*; empty vector control (blue) n = 100 (2 independent replicates), *glp-1(e2141)*; *daf-16* (RNAi) (red) n = 100 (2) and *glp-1(e2141)*; *daf-18* (RNAi) (green) n = 100 (2) when exposed to *X. nematophila*. (B) The effect of proteasomal complex genes identified as being integral for survival towards *X. nematophila* from genome wide analysis. Survival of *C. elegans glp-1(e2141)*; empty vector control (blue) n = 100 (2), *glp-1(e2141)*; *rpn-6.1* (RNAi) (red) n = 100 (2), *glp-1(e2141)*; *rpn-7* (RNAi) (green) n = 60 (2), *glp-1(e2141)*; *pbs-6* (RNAi) (purple) n = 100 (2), *glp-1(e2141)*; *rpn-10* (RNAi) (turquoise) n = 100 (2), *glp-1(e2141)*; *rpt-6* (RNAi) (orange) n = 100 (2) and *glp-1(e2141)*; *pbs-4* (RNAi) (light blue) n = 100 (2) when exposed to *X. nematophila*. (C) The effect of new genes identified as being integral for survival towards *X. nematophila* from genome wide analysis. Survival of *C. elegans glp-1(e2141)*; empty vector control (blue) n = 100 (2), *glp-1(e2141)*; T12G3.6 (RNAi) (purple) n = 100 (2), and *glp-1(e2141)*; *par-5* (RNAi) (orange) n = 60 (2) when exposed to *X. nematophila.*

From our microarray data we observed a significant overlap of our up-regulated gene set with known DAF-16 targets [51] (one-third of known DAF-16 targets upregulated in germline-ablated animals, p-value of overlap  = 5.23E-07, Figure 3B). DAF-16 is a key transcriptional regulator of insulin signaling output which is an important regulator of lifespan and innate immunity under various contexts including germline-ablation [1], [2], [25]–[28]. We also observed transcriptional up-regulation of *daf-16* itself (Table S2), and consistent with its reported role in pathogen resistance and longevity assurance [1], [2], [25]–[28], its knock-down by RNAi reduced the survival on pathogen in our assays (Figure 4A, see Table S8 for summary statistics). The PTEN homolog lipid phosphatase DAF-18 also acts downstream to the Insulin receptor DAF-2 and the PI3-Kinase AGE-1 to regulate the activity of DAF-16 [52]. Consistent with its lifespan-promoting role [53], we found it to be highly up-regulated in our microarray results. We further found that the RNAi based knock-down of *daf-18* resulted in reduced survival of *glp-1* mutants on *X. nematophila* (Figure 4A, see Table S8 for summary statistics). Thus, we demonstrate for the first time the requirement of *daf-18* for the extended lifespan and pathogen resistance in a germline-deficient background.

### Proteasomal components are essential for pathogen resistance of long-lived animals

We observed significant up-regulation of “proteasomal core complex activity” (GO analysis Table-S6(C)) as well transcriptional up-regulation of almost all components of the proteasomal complex (27 out of 32 genes from gene families *pas*-, *pbs*-, *rpn*- and *rpt*-, see Tables S5 and S7), suggesting that enhanced proteasomal activity is a key contributor to longer lifespan of germline-deficient animals, a hypothesis consistent with previously reported results [22], [49]. Indeed, many of the proteasome related genes in our dataset are already known to reduce lifespan of *glp-1* mutants [22], [49]. Therefore, we tested their role in increased pathogen resistance of *glp-1* mutants, and found that RNAi of these genes leads to a significant reduction in pathogen survival as well (Figure 4B). While the role of proteasomal genes in lifespan extension of germline-deficient mutants has been reported before, our results indicate that almost all of these components are transcriptionally up-regulated simultaneously in ablated animals, and that this up-regulation requires the presence of the gonadal tissue. In addition, we show that all of these genes are also essential for defense against pathogens.

### Discovery of new genes with role in pathogen resistance and lifespan extension in germline deficient *C. elegans*

In addition to the genes discussed above, we also found a significant reduction in pathogen resistance and lifespan upon RNAi of other genes not previously known to be involved with lifespan or innate immunity. Specifically, we could show that when the 14-3-3 protein *par-5* is knocked down via RNAi, both survival on *X. nematophila* and lifespan are significantly impaired in *glp-1(e2141)* animals (Figure 4C, Figure S2, summary statistics in Table S8, S9) but not in un-ablated *C. elegans* WT animals (Figure S3, Table S10). PAR-5 is known to regulate DAF-16 activity and lifespan [24], [54], but it has so far never been implicated in affecting survival against pathogens. We also identified an uncharacterized gene T12G3.6 (Figure 4C, Figure S2, Table S8,S9) which has been predicted to be involved in germline development in *C. elegans* [55] and is known to be induced in response to the fungal pathogen *Drechmaria coniospora* [56]. Our results show a gonad-dependent up-regulation of this gene in germline-ablated animals, and a requirement for this gene in mediating the increase in lifespan and pathogen survival of germline-ablated *C. elegans* (Figure 4C, Figure S2, Table S8,S9).

It is important to note that while the RNAi based knock-down of some of these candidate genes in germline-ablated animals leads to a decrease in survival on *X. nematophila* as well as lifespan, the lifespan of un-ablated *C. elegans* remains unaffected upon the RNAi of these genes (Figure S3, Table S10). This suggests a specific role for these genes in germline-ablation mediated lifespan extension, and serves as a functional validation of our system-wide approach. However, some of the candidate genes that reduced survival on *X. nematophila* and lifespan in germline-ablated animals, also reduced survival of unablated *C. elegans* e.g. *gna-2* and C14B1.2 (data not shown). Therefore, these genes seem to affect the general viability of the worm and were hence not further investigated. From these results we conclude that some of the genes observed to be upregulated in our microarray profile are specifically required for mediating the effects of germline-ablation on lifespan and immunity, while other genes might be required for general viability and normal development. Similar tests on the rest of the upregulated genes will therefore be needed to identify genes with specific roles in germline-mediated lifespan regulation.

### Evolutionary conservation between *C. elegans* and *P. pacificus*

Comparison of similar biological processes in different species and model systems is helpful in identifying evolutionary trends and conserved components. To the best of our knowledge, the only study to utilize whole genome microarrays to understand the patterns and processes involved with germline deficient lifespan extension and bacterial resistance has been carried out in *P. pacificus* [2]. In this study whole genome microarrays comparisons between germline-ablated long-lived *P. pacificus* cultured either on *Escherichia coli* OP50 or on the nematode pathogen *S. marcescens* showed that the expression profiles of long-lived animals was remarkably similar to pathogen resistant animals, implying that in both scenarios similar stress response pathways are activated that contribute to the increased survival [2].

**Figure 5:**
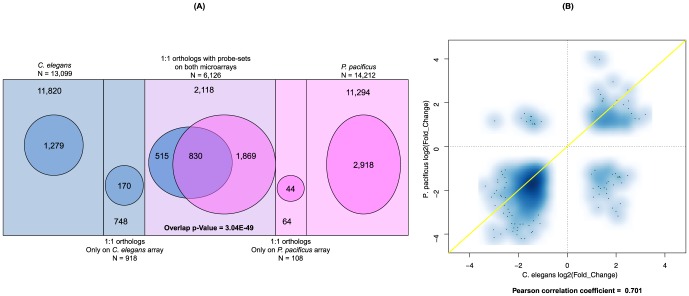
Evolutionary conservation of genes differentially regulated upon germline ablation in *C. elegans* compared to *P. pacificus*. (A) Significant overlap between germline-regulated genes in *C. elegans* and *P. pacificus* [2]. Fisher's exact test p-value  = 3.04E-49. (B) Most of the germline regulated genes in *C. elegans* and *P. pacificus* [2] also show similar expression patterns (Pearson correlation coefficient  = 0.7).

Interestingly, a significant proportion of these germline-regulated genes also show a significant overlap and similar expression patterns in both *C. elegans* and *P. pacificus* (Figure 5). These findings highlight a great extent of evolutionary conservation in this biological process across the two species, which shared a common ancestor 250–400 million years ago [57]. This overlap is even more remarkable given the fact that our previous gene expression studies comparing *C. elegans* and *P. pacificus* in other contexts revealed relatively more diverged expression profiles [42], [47]. Hence, the significant overlap between germline-regulated genes in the two species suggests a deeper conservation of underlying biological processes such as insulin signaling and proteasome function.

## Discussion

The presence of gonadal tissue is essential for the increased lifespan observed in germline deficient *C. elegans* [1]. Although many genes have been identified as being essential in regulating *C. elegans* lifespan in germline deficient animals e.g. *daf-16* and *daf-12* [1], [14]–[24], the gamut of genes and processes regulated by the gonad has not been studied systematically at a whole genome level in this context before [58]. Here we first show that the gonad tissue is also needed for enhanced pathogen resistance phenotype of germline-ablated *C. elegans*. Next, using a microarray based approach we have identified a set of candidate genes that are regulated in a gonad dependent manner in germline-ablated animals, and via RNAi based knock-downs demonstrated the requirement for some of these genes in both longer lifespan as well as enhanced pathogen resistance. These data will therefore be useful in further dissection of genetic components of gonad dependent longevity signals. Furthermore, by comparing our data to germline-ablated *P. pacificus* [2] we demonstrate that many of these genes and processes that increase lifespan and bacterial resistance remain highly conserved during evolution. Specifically, we could show that upon germline-ablation, both *P. pacificus* and *C. elegans* upregulate genes involved in proteasomal complexes (*pbs*-, *rpt*-, *rpn*- gene families), chaperon activity (*cct*-gene family), fatty-acid desaturases (*fat*-gene family) and eukaryotic translation elongation factors, and insulin signaling (*daf-18*, *age-1* and *daf-16*), which together potentially contribute to their increased lifespan and survival on pathogens [2].

We show that the genes such as *daf-2*, *daf-16*, *daf-12*, *tcer-1*, *mir-7.1* and *nhr-80*, which were originally identified as being essential for the increase in lifespan of germline-deficient *C. elegans* are also required for their enhanced resistance to bacterial pathogens. Next, by combining cell ablation with microarray experiments and gene knockdown analysis in germline deficient *C. elegans* we have built a genome-wide catalogue of about 3,440 candidate genes that are potentially essential for higher lifespan and pathogen resistance of these Z(2,3) deficient animals as compared to the short-lived and pathogen-susceptible Z(1,4) deficient animals. Using RNAi-mediated knockdowns of a subset of 150 genes from the microarray data, we tested for their requirement in lifespan extension and pathogen resistance across three different conditions (*glp-1* mutants on pathogen, *glp-1* mutants on *E. coli*, wild-type animals on *E. coli*). We could thus validate the specific requirement of 10 genes out of the 150 candidate genes tested for lifespan extension and pathogen resistance, including the PTEN tumor repressor *daf-18*, proteasomal complex genes (*rpn-6.1*, *rpn-7*, *rpn-9*, *rpn-10*, *rpt-6*, *pbs-3* and *pbs-6*) and two novel genes (*par-5* and T12G3.6). Among the novel candidates tested, although we observed a reduction in resistance to bacterial pathogens in 3 genes (T12G3.6, *gna-2*, C14B1.2), only T12G3.6 was found to be specific to lifespan extension of germline-ablated animals, while the knock-down of other genes also reduced the lifespan of unablated *C. elegans*. Hence, similar tests on more candidate genes will be required to identify novel genes specifically involved in lifespan extension. The seemingly low proportion of novel candidates that could be validated from microarray data is not entirely unusual. For example, in a previous study of *C. elegans* response to *P. aeruginosa*, 38 genes were identified via microarray analysis but none of them enhanced susceptibility upon RNAi [59]. In another similar study, RNAi of only 9% of the induced genes and 11% of transcriptionally repressed genes compromised pathogen resistance of *C. elegans* when fed *P. aeruginosa* PA14 [60]. The reasons for this could include functional redundancy between multiple genes, or inefficient knockdown of genes via RNAi [59]. In summary, apart from the genes we could test here, we believe that our results present a useful resource for selecting candidate genes for future screens of genetic basis of increased lifespan and survival in germline-ablated animals.

## Materials and Methods

### Nematode and bacteria strains

*C. elegans* N2, *daf-2(e1370)*, *daf-16(m26)*, *daf-16(m27)*, *daf-16(m86)*, *tcer-1(tm1452)*, *nhr-80(tm1011)*, *daf-12(m20)*, *mir-71(n4115)* and *glp-1(e2141)* were obtained from the *Caenorhabditis* Genetic Stock Centre, U.S. and maintained on 5 cm NGM agar plates spotted with *E. coli* (strain OP50) at 20°C. *X. nematophila* strain XN2 was a gift from Becker Underwood, U.K. and was maintained on LB plates.

### Survival assays and analysis

*X. nematophila* was grown in LB at 30°C overnight in a shaking incubator. The following day 100 µl were spread evenly onto pre-dried 5 cm NGM plates and incubated overnight at 30°C. Batches of 10 ablated or un-ablated *C. elegans* were picked onto *X. nematophila* plates and monitored daily for survival. Worms that failed to respond to a touch of the worm-pick were considered dead. Survival of ablated or un-ablated *C. elegans* fed on *X. nematophila* or *E. coli* OP50 was compared using Kaplan meier curves and the Log Rank test using the OASIS web-tool [61] (Online Application for the Survival Analysis of Lifespan Assays Performed in Aging Research, http://sbi.postech.ac.kr/oasis).

### Cell ablation and RNA collection

Wild type or mutant *C. elegans* L1 stage larvae were picked into 2.8 µl PBS on an agar pad containing 1 mM NaN_3_. Ablations would take place within 1 hour of hatching at 20°C. After ablation nematodes were stored at 20°C and successful ablation was verified 48 hours later. Un-ablated *C. elegans* were grown in parallel and served as controls. For microarrays 20 young-adult *C. elegans* (either Z(2,3)-ablated, or Z(1,4)-ablated, or un-ablated) were picked onto 5 separate NGM plates spotted with *E. coli* OP50 and incubated at 25°C for 4 hours. Nematodes were then picked into 500 µl of Trizol and stored at −80°C until further analysis. The treatments therefore included (i) Z(2,3)-ablated *C. elegans* fed *E. coli* OP50 (ii) Z(1,4)-ablated *C. elegans* fed *E. coli* OP50, and (iii) un-ablated *C. elegans* fed *E. coli* OP50. Developmental time course of ablated and un-ablated *C. elegans* fed *E. coli* OP50 was the same. Each treatment consists of approximately 100 animals and was repeated three times.

### Microarray experiments and data analysis

*C. elegans* microarrays from Agilent Technologies were used in a two-color format, and the experiments and data analysis was performed essentially as described before [42], [47], with three biological replicates per treatment condition. The first set of hybridizations compared Z(2,3)-ablated worms against un-ablated wild-type *C. elegans*, while the second set of hybridizations compared gonad Z(1,4)-ablated worms against un-ablated wild-type *C. elegans*. The differential expression between germline-ablated versus gonad-ablated animals was computed via the functions “makeContrasts” and “contrasts.fit” in the “limma” package [62] in R/Bioconductor [63]. The raw and normalized data for all the experiments from this publication have been deposited in a MIAME compliant format at NCBI's Gene Expression Omnibus database (http://www.ncbi.nlm.nih.gov/geo/) under accession numbers GSE44702 and GSE44703.

Gene ontology analysis was carried out using the R package topGO [64]. List of genes whose knock-down leads to the phenotype “shortened life span”, and the list of members of each gene family in *C. elegans*, were extracted from WormMart version WS220 (http://caprica.caltech.edu:9002/biomart/martview/). List of germline-enriched genes [46], [48], and DAF-16 targets [51] was extracted from WormBase as the following “Expression Clusters”: {cgc6390}:oogenesis-enriched, {cgc6390}:herm_sex-enriched, {cgc6390}:mixed_oogenesis-somatic, WBPaper00037611:RNP-8-associated, WBPaper00037611:GLD-2-associated, and {cgc5976}:class_1.

All overlap and enrichment analysis was statistically tested for significance using Fisher's exact test.

### RNAi screening of longevity/immunity genes in *C. elegans*

RNAi clones from the Ahringer library (Source BioScience LifeSciences, UK), which were annotated as “Correct”, were grown at 37°C in LB with 50 µg/ml ampicillin overnight. One hundred microlitres of overnight culture were then spread on NGM RNAi agar plates containing 25 µg/ml carbenicillin and 1 mM IPTG and allowed to induce overnight at 20°C. The next day 2-3 *glp-1*(*e2141*) young-adults were picked on to 3 plates per genotype and stored at 15°C. When numerous eggs were laid, the plates were shifted to 25°C to induce germline depletion in these progeny. After about 2.5 days, 60 to 100 young-adult nematodes were picked onto 3 to 5 plates containing *X. nematophila* and their survival was monitored daily (immunity assays). For longevity assays the same procedure was repeated except that the young adult worms continued to be reared on the respective RNAi clone bearing bacteria on NGM RNAi plates. We used two positive RNAi controls against *unc-22* and *dpy-9*, to ensure that RNAi was efficient.

## Supporting Information

Figure S1 **Schematic of RNAi screen for genes involved with** ***C. elegans glp-1(e2141)*** **survival against** ***X. nematophila*** **.**
(TIF)

Figure S2 **The new genes identified as being integral for survival towards** ***X. nematophila*** **are also essential for lifespan.** Lifespan analysis of *C. elegans glp-1(e2141)*; empty vector control (RNAi) (blue) n = 100 (2 independent replicates), *glp-1(e2141)*; T12G3.6 (RNAi) (green) n = 100 (2), and *glp-1(e2141); par-5* (RNAi) (red) n = 90 (2) when fed *E. coli* OP50.
(PDF)

Figure S3 **The effect of RNAi knock down of genes identified on normal lifespan of** ***C. elegans*** **wild type.** Lifespan analysis of *C. elegans* WT; empty vector control (RNAi) (blue) n = 60 (2), *C. elegans* WT; T12G3.6 (purple) n = 63 (2), and *C. elegans* WT; *par-5* (RNAi) (red) n = 60 (2) when fed *E. coli* OP50.
(PDF)

Table S1 **Summary lifespan statistics of** ***C. elegans*** **ablation experiments monitoring survival when fed** ***X. nematophila*** **.** Mean survival and standard errors for all mutants tested, and p-values from log-rank test assessing significance of survival compared to *C. elegans* Z(2,3)-ablated.
(XLSX)

Table S2 **Genes differentially regulated in germline-ablated animals.** Comparison of germline Z(2,3)-ablated animals against intact wild-type controls.
(XLSX)

Table S3 **Significant overlap between known germline enriched genes and genes down-regulated in our microarray comparison of germline-ablated versus un-ablated** ***C. elegans*** **.**
(XLSX)

Table S4 **Genes differentially regulated in gonad-ablated animals.** Comparison of germline Z(1,4)-ablated animals against intact wild-type controls.
(XLSX)

Table S5 **Genes differentially regulated in long-lived** ***C. elegans*** **in a gonad-dependent manner.** Comparison of Z(2,3)-ablated animals against gonad Z(1,4)-ablated animals. Components of the proteasomal complex are marked by the value "yes" and highlighted in green rows.
(XLSX)

Table S6 **Gene Ontology (GO) class enrichment analysis in differentially regulated in long-lived animals.**
(XLSX)

Table S7 **Gene families enriched in the set of differentially regulated genes in long-lived animals.**
(XLSX)

Table S8 **Summary lifespan statistics of** ***C. elegans glp-1(e2141)*** **mutants fed on** ***X. nematophila*** **, upon RNAi of selected genes identified from genome wide microarray experiments.** Mean survival and standard errors for all genes tested, and p-values from log-rank test assessing significance of survival compared to *C. elegans glp-1(e2141)* when fed empty vector control.
(XLSX)

Table S9 **Summary lifespan statistics of** ***C. elegans glp-1(e2141)*** **mutants upon RNAi of selected genes identified from genome wide microarray experiments.** Mean survival and standard errors for all genes tested, and p-values from log-rank test assessing significance of survival compared to *C. elegans glp-1(e2141)* when fed empty vector control.
(XLSX)

Table S10 **Summary lifespan statistics of** ***C. elegans*** **wild type upon RNAi of selected genes identified from genome wide microarray experiments.** Mean survival and standard errors for all genes tested and p-values from log-rank test assessing significance of survival compared to *C. elegans* wild type when fed empty vector control.
(XLSX)
