# Supplementary figures and images for: A Functional Genomic Screen for Evolutionarily Conserved Genes Required for Lifespan and Immunity in Germline-Deficient C. elegans
Source: PLoS One. 2014 Aug 5;9(8):e101970. doi: 10.1371/journal.pone.0101970 (PMC4122342; doi:10.1371/journal.pone.0101970)

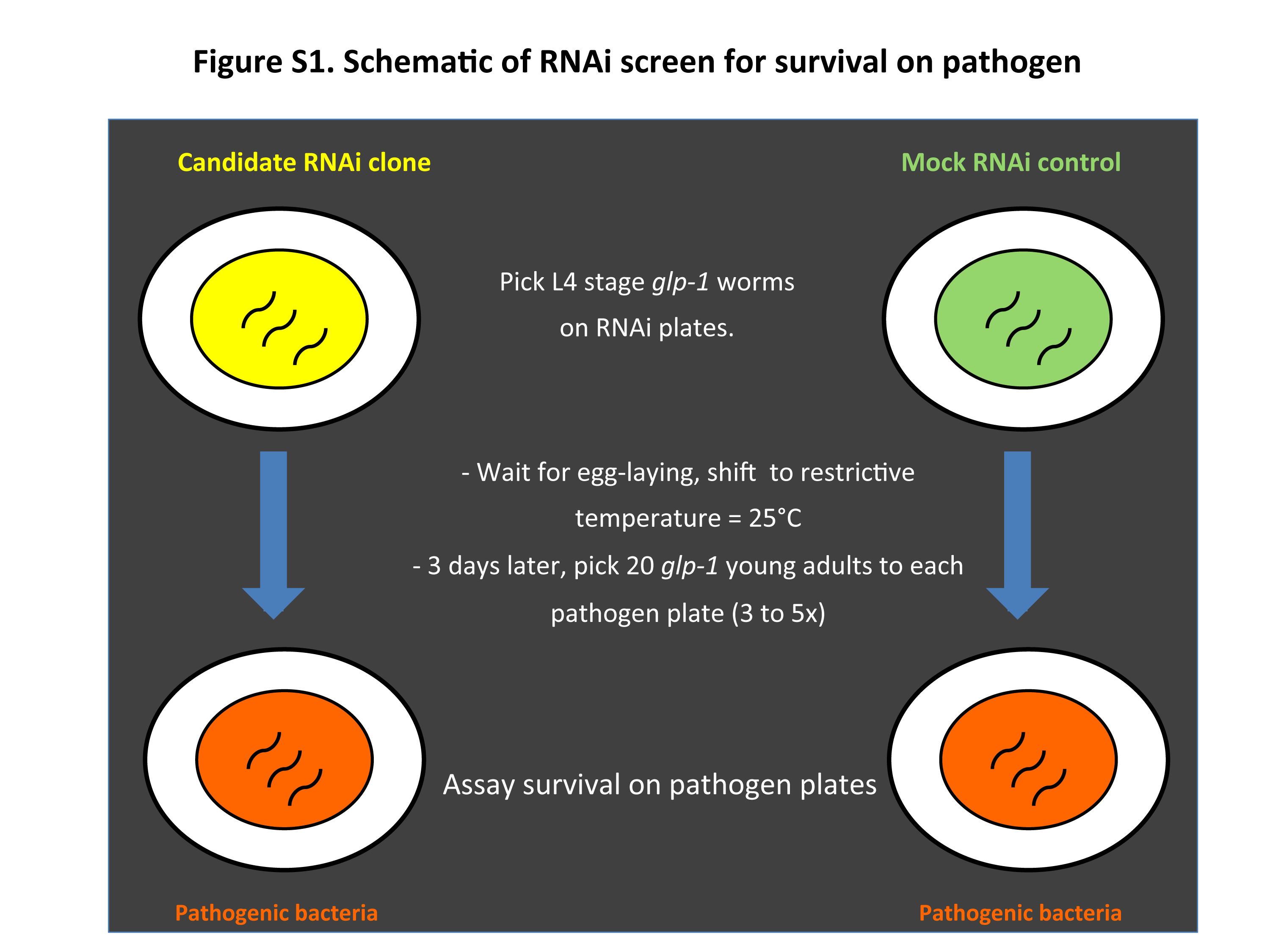

Supplement: Figure S1 — Schematic of RNAi screen for genes involved with C. elegans glp-1(e2141) survival against X. nematophila . (TIF) [file pone.0101970.s001.tif]
