# Supplementary material for: A Functional Genomic Screen for Evolutionarily Conserved Genes Required for Lifespan and Immunity in Germline-Deficient C. elegans
Source: PLoS One. 2014 Aug 5;9(8):e101970. doi: 10.1371/journal.pone.0101970 (PMC4122342; doi:10.1371/journal.pone.0101970)

Figure S2: The new genes identified as being integral for survival towards *X. nematophila* are also essential for lifespan. Lifespan analysis of *C. elegans glp-1(e2141);* empty vector control (RNAi) (blue) n = 100 (2 independent replicates), *glp-1(e2141);* T12G3.6 (RNAi) (green) n = 100 (2), and *glp-1(e2141); par-5* (RNAi) (red) n = 90 (2) when fed *E. coli* OP50.

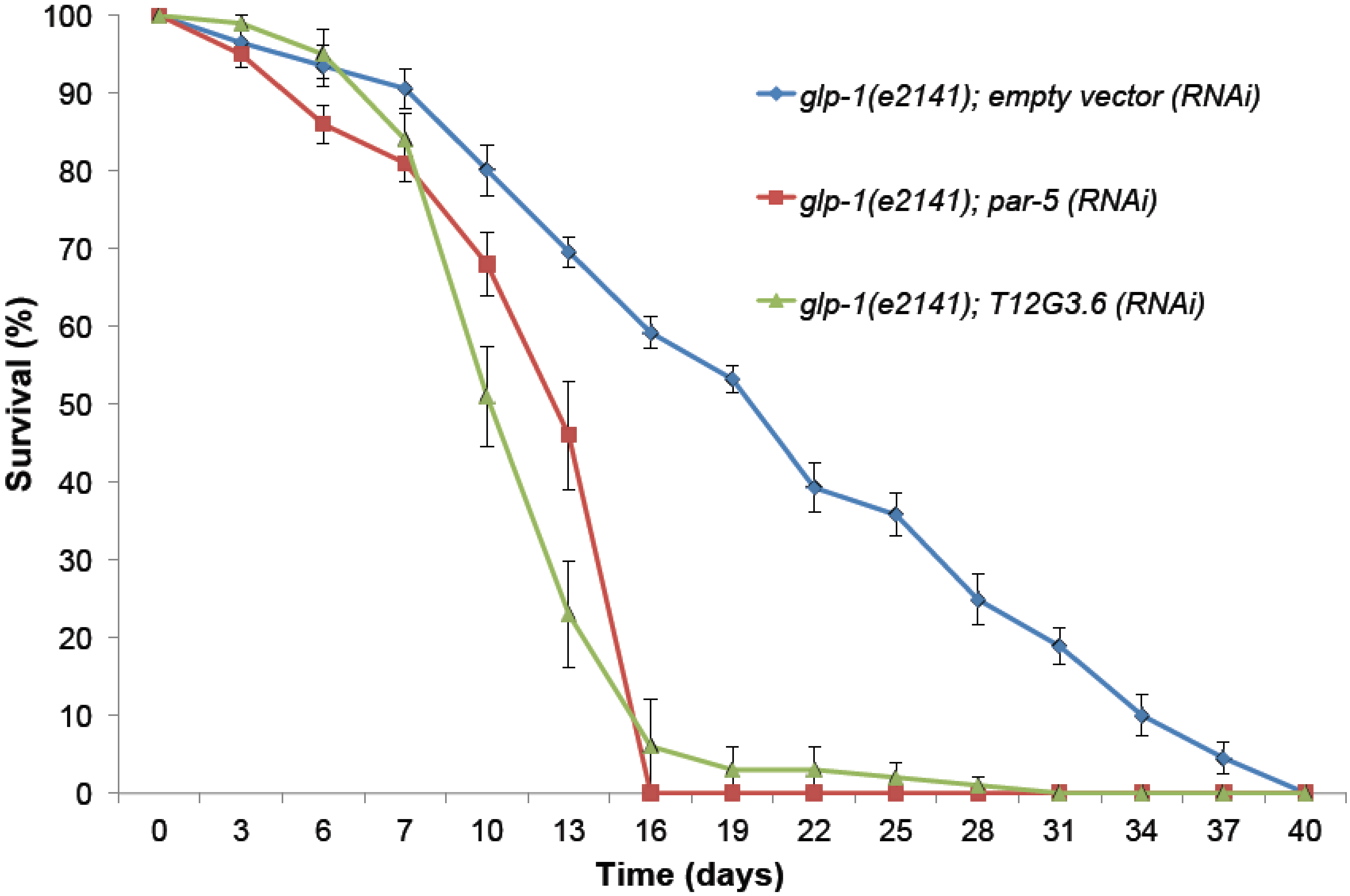

Supplement: Figure S2 — The new genes identified as being integral for survival towards X. nematophila are also essential for lifespan. Lifespan analysis of C. elegans glp-1(e2141); empty vector control (RNAi) (blue) n = 100 (2 independent replicates), glp-1(e2141); T12G3.6 (RNAi) (green) n = 100 (2), and glp-1(e2141); par-5 (RNAi) (red) n = 90 (2) when fed E. coli OP50. (PDF) [file pone.0101970.s002.pdf]
