# Supplementary material for: A Functional Genomic Screen for Evolutionarily Conserved Genes Required for Lifespan and Immunity in Germline-Deficient C. elegans
Source: PLoS One. 2014 Aug 5;9(8):e101970. doi: 10.1371/journal.pone.0101970 (PMC4122342; doi:10.1371/journal.pone.0101970)

Figure S3: The effect of RNAi knock down of genes identified on normal lifespan of *C. elegans* wild type.

Lifespan analysis of *C. elegans* WT; empty vector control (RNAi) (blue) n = 60 (2), *C. elegans* WT*;* T12G3.6 (purple) n = 63 (2), and *C. elegans* WT*; par-5* (RNAi) (red) n = 60 (2) when fed *E. coli* OP50.

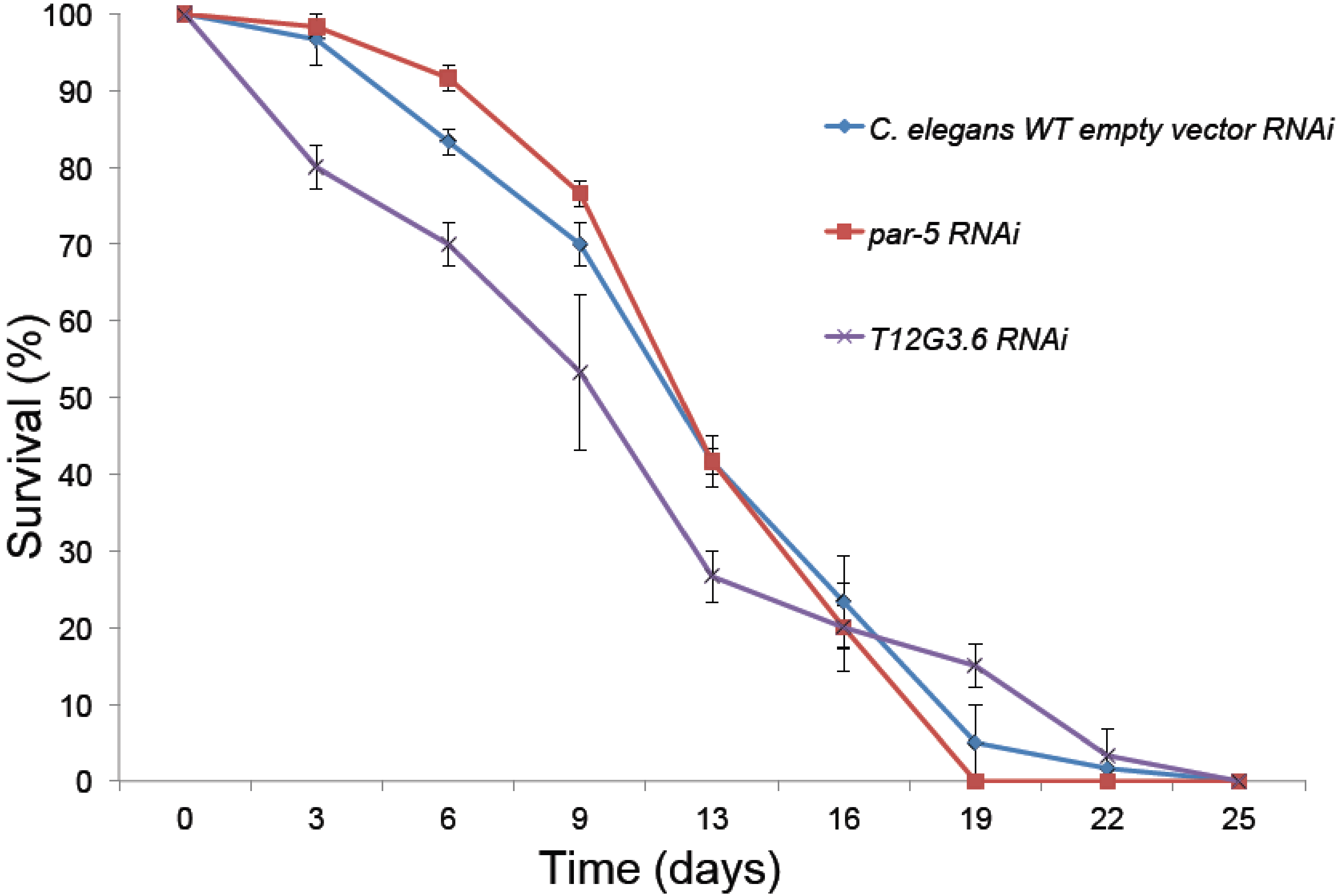

Supplement: Figure S3 — The effect of RNAi knock down of genes identified on normal lifespan of C. elegans wild type. Lifespan analysis of C. elegans WT; empty vector control (RNAi) (blue) n = 60 (2), C. elegans WT; T12G3.6 (purple) n = 63 (2), and C. elegans WT; par-5 (RNAi) (red) n = 60 (2) when fed E. coli OP50. (PDF) [file pone.0101970.s003.pdf]
